# Dark chocolate or tomato extract for prehypertension: a randomised controlled trial

**DOI:** 10.1186/1472-6882-9-22

**Published:** 2009-07-08

**Authors:** Karin Ried, Oliver R Frank, Nigel P Stocks

**Affiliations:** 1 Discipline of General Practice, The University of Adelaide, Adelaide, SA 5005, Australia

## Abstract

**Background:**

Flavanol-rich chocolate and lycopene-rich tomato extract have attracted interest as potential alternative treatment options for hypertension, a known risk factor for cardiovascular morbidity and mortality. Treatment of prehypertension (SBP 120–139/DBP 80–89 mmHg) may forestall progression to hypertension. However, there has been only limited research into non-pharmacological treatment options for prehypertension. We investigated the effect of dark chocolate or tomato extract on blood pressure, and their acceptability as an ongoing treatment option in a prehypertensive population.

**Methods:**

Our trial consisted of two phases: a randomised controlled three-group-parallel trial over 12 weeks (phase 1) followed by a crossover of the two active treatment arms over an additional 12-week period (phase 2). Group 1 received a 50 g daily dose of dark chocolate with 70% cocoa containing 750 mg polyphenols, group 2 were allocated one tomato extract capsule containing 15 mg lycopene per day, and group 3 received one placebo capsule daily over 8 weeks followed by a 4-week washout period. In phase 2 the active treatment groups were crossed over to receive the alternative treatment. Median blood pressure, weight, and abdominal circumference were measured 4-weekly, and other characteristics including physical activity, general health, energy, mood, and acceptability of treatment were assessed by questionnaire at 0, 8 and 20 weeks. We analysed changes over time using a linear mixed model, and one time point differences using Kruskal-Wallis, Fisher's-Exact, or t-tests.

**Results:**

Thirty-six prehypertensive healthy adult volunteers completed the 6-month trial. Blood pressure changes over time within groups and between groups were not significant and independent of treatment. Weight and other characteristics did not change significantly during the trial. However, a marked difference in acceptability between the two treatment forms (chocolate or capsule) was revealed (p < 0.0001). Half of the participants allocated to the chocolate treatment found it hard to eat 50 g of dark chocolate every day and 20% considered it an unacceptable long-term treatment option, whereas all participants found it easy and acceptable to take a capsule each day for blood pressure.

**Conclusion:**

Our study did not find a blood pressure lowering effect of dark chocolate or tomato extract in a prehypertensive population. Practicability of chocolate as a long-term treatment option may be limited.

**Trial registration:**

Identifier: ACTRN12609000047291

## Background

Prehypertension (systolic blood pressure (SBP) 120–139 mm Hg, diastolic blood pressure (DBP) 80–89 mm Hg) is considered a precursor of hypertension and has been associated with an increased risk of up to 3.5 times in cardiovascular morbidity and mortality later in life [1-6]. Treatment of prehypertension with an angiotensin-receptor blocker for 2 years and follow-up at 4 years resulted in a significant 27% reduction in new-onset hypertension in the treated group compared to placebo [4].

Lifestyle modifications including dietary approaches have been recommended for the management of prehypertension [1,7]. Because alternative and complementary treatment options for blood pressure have become increasingly popular [8,9], we investigated whether dark chocolate or tomato extract would be effective or acceptable treatment options for prehypertension.

Polyphenols, in particular flavanols, in chocolate have been associated with the formation of endothelial nitric oxide leading to vasodilation and lowering of blood pressure [10-12], whereas the blood pressure lowering effect of tomato extract has been linked to its high antioxidant content, in particular the carotenoid lycopene, and its free radical scavenging properties [13,14].

Chocolate has recently been a popular target in studies investigating dietary effects on blood pressure [15-27]. However, most studies were either of short duration (2 weeks) [18-23,27] or had no control group without cocoa [15] (same study reported also in [17]). In contrast, tomato extract as the sole treatment for blood pressure has been less widely researched [28], and only in a hypertensive population. To date, a limited number of controlled trials of longer than 2 weeks duration, in a prehypertensive population, and exploring the effect of chocolate on blood pressure have been conducted with conflicting results [16,24]. Moreover, to our knowledge, no study to date has compared the acceptability and practicability of different treatment modalities for high blood pressure or prehypertension.

## Methods

### Trial participants

**Figure 1:**
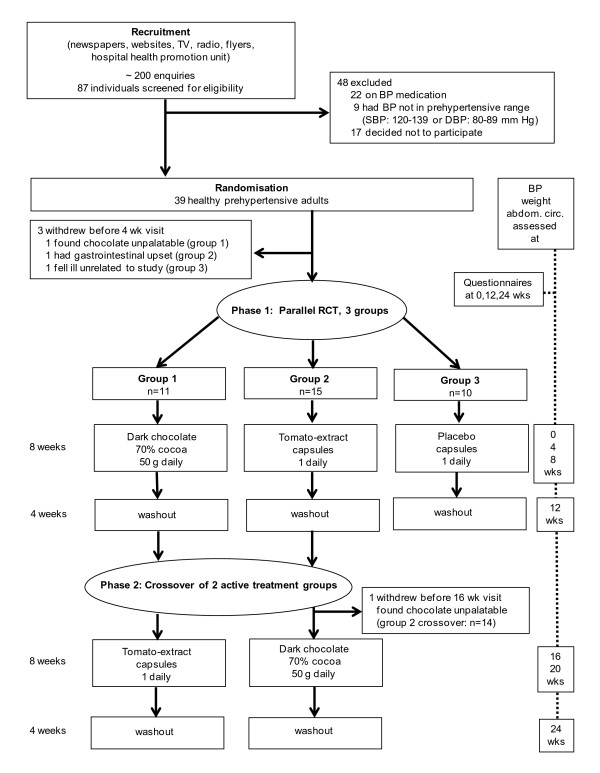
**Trial flow**.

The trial was conducted between June and December 2007 in Adelaide, South Australia. Recruitment started in February 2007 by advertising in local newspapers, the University websites, flyers around University noticeboards, a public hospital's health promotion unit, and items on local radio and evening television news programs. We received about 200 enquiries regarding the study, with 87 individuals filling in a screening questionnaire assessing eligibility criteria including recent blood pressure readings and availability for the 6-month study. Inclusion criteria were: 1. Two of either four systolic or diastolic blood pressure readings in the prehypertensive range (SBP 120–139 or DBP 80–89 mm Hg), as measured by our research nurse; 2. Not taking any antihypertensive prescription medication. Exclusion criteria were: 1. Diabetes mellitus; 2. Allergy to or intolerance of tomato or chocolate products. Forty-eight individuals did not meet inclusion criteria (n = 31) or decided not to participate (n = 17) including some who did not want to take the chance of not being randomised to the chocolate group. The remaining 39 healthy volunteers, aged between 22 and 73 years, provided written consent and were allocated to one of three treatment arms (Figure 1).

The study was approved by the Human Research Ethics Committee of The University of Adelaide.

### Trial design

Our trial consisted of two phases: a three group parallel randomised controlled trial (phase 1), followed by a crossover of the two active treatment arms (phase 2). The duration of each study arm was 12 weeks, consisting of an 8-week intervention period followed by a 4-week washout period. Participants were randomly allocated to the chocolate (group 1), tomato extract (group 2), or placebo control (group 3) group by permuted block randomisation using the SAS 9.1 software package [29]. Participants remained in these three groups for 12 weeks (phase 1), followed by the two active treatment groups (group 1 and 2) being crossed over to the other active treatment group for an additional 12 weeks (phase 2). The control group (group 3) ceased participation after phase 1 (at 12 weeks) (Figure 1).

Over an 8-week period, group 1 participants were allocated a 50 g daily dose (half of a 100 g block) of commercially available dark chocolate with 70% cocoa, containing 750 mg of polyphenols (Haighs Chocolates, Adelaide, South Australia [30]); group 2 was allocated one commercially available tomato extract capsule per day containing 15 mg lycopene (Lyc-o-Mato^® ^Tomato Lycopene Complex capsules, LycoRed Ltd, Beer Sheva, Israel, equivalent to four to five medium size tomatoes) [31]; and group 3 was allocated one placebo capsule per day (matched to tomato extract capsules by colour, odour and size, and containing mainly soy oil). Polyphenol content of the trial chocolate was analysed by the Australian Government National Measurement Institute using the colorimetric Folin-Ciocalteu assay. The trial chocolate contained 15 mg/g of total polyphenols, equivalent to 395 umol/g ORAC (oxygen radical absorbance capacity).

To conceal allocation from investigators, trained staff not involved in trial design and analysis handed out intervention packs to participants. Blinding of participants to chocolate was impractical, however blinding of participants in the capsule groups was achieved by identical packaging of active tomato extract and placebo capsules.

Blood pressure, weight and abdominal circumference were measured every 4 weeks by a trained research nurse, and questionnaires assessing physical activity, smoking status, alcohol intake, general health, mood and energy levels, habitual chocolate and tomato product intake, ease and acceptability of treatment, were administered at baseline and after each intervention period (at 0, 8 and 20 weeks). The questionnaire consisted of 5-point Likert-scales, and closed and open ended questions, and can be obtained from the corresponding author.

Habitual lycopene intake at baseline was estimated using summary values of lycopene content in tomato products based on five databases by Porrini M and Riso P [32]. Participants were counselled to maintain their usual diet and physical activity during the trial. Compliance and any additional chocolate or tomato product intake was assessed using participants' daily diary entries. A follow-up questionnaire was administered 3 months after the trial to assess chocolate and tomato consumption habits.

### Blood pressure monitoring

Blood pressure was assessed by a trained research nurse using a digital calibrated sphygmomanometer with appropriately sized cuffs (Omron HEM-907, JA Davey Pty Ltd). Participants rested for 5 minutes before four blood pressure readings were taken in intervals of 3 minutes in alternate upper arms in a seated position. Median SBP and DBP at one time point were used for analysis to minimise the effect of blood pressure variability.

### Sample size and data analysis

On a population level a reduction of 4 to 5 mm Hg in SBP and 2 to 3 mm Hg in DBP has been estimated to reduce the risk of cardiovascular morbidity and mortality by up to 20 per cent [33]. We calculated that a sample size of 60, with 20 individuals in each group, would allow detection of this difference at a power of 0.75 with 95% confidence assuming a correlation of 0.5 among repeated measures.

As random number generation was based on a sample size of 60, which we were unable to match in practice, numbers allocated to each of the three groups were slightly disproportionate. Smaller group sizes between n = 10 and 15 in study 1 (three group parallel RCT) reduced the power to 0.5 for detecting a difference of 4.5 mm SBP and 2.5 mm DBP, while reductions of 5–6 mm SBP and 3–4 mm DBP would retain the power at 0.8. However, larger group sizes in the crossover analysis (chocolate groups n = 26, capsule groups n = 36) assessing acceptability of the two treatment forms led to a reasonable effect size > 0.9.

Analyses were performed using SPSS version 15.0 and SAS version 9.1 [29,34]. Between-group differences at one time point were assessed by Kruskal-Wallis test (continuous variables) and Fisher's-Exact test (binominal variables). A linear mixed model was used to assess within-group and between-group differences of blood pressure over time. Multiple comparisons were adjusted by Holm's Stepdown Bonferroni procedure. P-values < 0.05 were considered statistically significant. Blood pressure changes over time were analysed separately for phase 1 (parallel three group RCT) and phase 2 (two group crossover). Adequate washout between studies was assumed. Acceptability of two treatment forms (chocolate or capsule) was analysed by student t-test.

## Results

### Baseline characteristics

We collected baseline characteristics of all 39 randomised prehypertensive individuals. However, three participants withdrew before the second time point for data collection scheduled at week 4. All comparisons were subsequently based on the thirty-six remaining participants. At baseline, groups did not differ in anthropometric measures, habitual chocolate or tomato product intake, physical activity, alcohol intake, general health, energy and mood levels. None of the participants smoked. A significantly higher proportion of participants in the chocolate and tomato extract groups took daily fish oil and/or garlic supplements than in the control group (Table 1). As there is evidence to suggest that fish oil or garlic supplementation may have a blood pressure reducing effect in hypertensive individuals [35,36], dietary supplementation was subsequently included as a binominal covariate in the analyses of blood pressure. While median SBP and DBP at baseline did not differ statistically between groups, observed differences were clinically relevant, and were therefore included as covariates in the linear mixed model analyses of blood pressure changes over time.

**Table 1:** Baseline characteristics

	**Mean (SD)**			
**Characteristics**	**Dark chocolate**	**Tomato extract**	**Control**	**P-value^a^**

	n = 11	n = 15	n = 10	

Age, years	48.8 (12.2)	51.2 (12.1)	57.9 (13.4)	.11

Weight, kg	80.3 (17.7)	74.0 (10.3)	75.9 (13.7)	.63

Body mass index	26.9 (6.2)	26.2 (3.1)	26.2 (5.2)	.87

Abdominal circumference, cm	85.7 (9.5)	87.5 (11.5)	82.2 (9.3)	.65

Median systolic blood pressure, mm Hg	135.1 (12.5)	128.2 (11.4)	135.7 (12.4)	.18

Median diastolic blood pressure, mm Hg	83.6 (10.5)	79.1 (7.5)	77.8 (8.6)	.34

Self-reported...				

Dark chocolate intake, g/wk	32.5 (55.0)	70.9 (166.9)	59.0 (61.3)	.43

Milk chocolate intake, g/wk	60.0 (120.2)	73.7 (102.7)	99.0 (172.1)	.42

Tomato product intake, d/wk	3.1 (1.7)	4.5 (1.4)	3.1 (1.9)	.07

Estimated habitual lycopene intake (only subjects ≥ 105 mg/wk)^c^	211 (74), n = 2	194 (16), n = 2	210 (0), n = 1,	not calculated

Moderate physical activity ≥ 30 min, d/wk^d^	2.9 (2.0)	3.8 (2.5)	3.3 (2.5)	.65

Alcohol intake, times/wk	1.4 (1.7)	2.6 (2.3)	2.9 (2.8)	.40

Standard drinks/wk	5.2 (6.6)	3.3 (3.0)	8.1 (11.1)	.84

Smoking, cigs/d	0	0	0	1

5-point Likert scales				

General health^e^	2.3 (0.9)	2.0 (0.8)	2.2 (0.8)	.58

Energy levels^f^	2.5 (0.8)	2.3 (1.0)	2.3 (0.8)	.65

Mood^g^	2.1 (0.5)	2.1 (0.7)	2.0 (0.7)	.92

Bivariate variables				

Gender				

Male	7	7	5	0.7^b^

Female	4	8	5	

Dietary supplement intake				

If fish oil or garlic = yes	4	9	1	.04^b^*

no	7	6	9	

^a ^P-values are calculated by Kruskal-Wallis Test or ^b ^Fisher's-Exact Test

^c ^Intake of one tomato extract capsule per day equals 105 mg lycopene per week.

^d ^Moderate physical activity, e.g. brisk walk, cycling at regular pace, doubles tennis, swimming

^e ^5-point scale with 1 = excellent, 2 = very good, 3 = good, 4 = fair, 5 = poor

^f ^5-point scale with 1 = very energetic, 2 = moderately energetic, 3 = somewhat energetic, 4 = fatigued, 5 = very fatigued

^g ^5-point scale with 1 = very happy, 2 = happy, 3 = up and down, 4 = unhappy, 5 = very unhappy

* p < 0.05 indicates a significant difference between groups

### Withdrawals and compliance

As mentioned above three individuals withdrew after baseline assessment but before the 4-week assessment. In addition, one individual withdrew after crossover to study 2 (after 12 wks but before the 16 wk assessment). Two individuals withdrew when allocated to a chocolate group (one in phase 1, one in phase 2), both indicating that they found the dark chocolate unpalatable (Figure 1). One individual allocated to the tomato extract capsules reported gastrointestinal upset and one individual allocated to the control group withdrew due to illness apparently unrelated to our trial. In total, 36 participants completed phase 1, twenty-five completed phase 2, and attended all monitoring sessions, respectively. Self-reported diary entries indicated excellent compliance with the study protocol and consumption of provided chocolate or capsules. Additional chocolate or tomato product consumption during the intervention and washout periods was negligible (data not shown).

### Outcomes

#### Blood pressure

**Figure 2:**
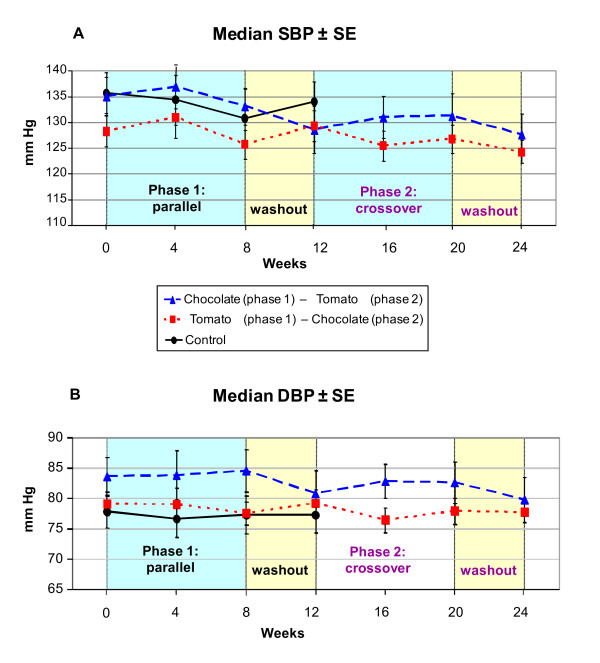
**Median systolic (A) and diastolic (B) blood pressure ± standard error (SE), during phase 1 (three group parallel RCT, 12 weeks) and phase 2 (crossover of active treatment groups, 12 weeks)**.

Observed blood pressure changes over time were inconsistent and independent of treatment (Figure 2). No significant blood pressure changes over time within groups or differences between groups were obtained (Table 2). Median SBP values remained within the prehypertensive range throughout the trial (120–139 mm Hg), while median DBP values stayed within the prehypertensive and upper normal range (75–89 mm Hg) (Table 2). There was a trend towards median systolic blood pressure reduction in the two active treatment groups over the 24 weeks of the trial, but this trend was independent from the treatment including the washout phases [p = 0.058; med SBP reduction (SE) in group 1: -7.5 (5.3) mm Hg; in group 2: -4.0 (4.7) mm Hg].

**Table 2:** Median BP ± SE (SD)

**Median SBP ± SE (SD)**											
		**Phase 1**					**Phase 2**				

	**N**	**Parallel RCT, 3 groups**					**Crossover of two active groups**				

**Group**		**wk 0**	**wk 4**	**wk 8**	**washout wk 12**	**P-value within group over time wk 0–12**	**Group**	**wk 16**	**wk 20**	**Washout wk 24**	**P-value within group over time wk 0–24**

**Chocolate**	11	135.0 ± 3.8 (12.5)	136.9 ± 4.3 (14.1)	133.1 ± 3.5 (11.7)	128.6 ± 4.5 (15.0)	.23	**Tomato**	131.0 ± 4.1 (13.6)	131.2 ± 4.3 (14.2)	127.6 ± 3.9 (13.0)	.57

**Tomato**	15	128.2 ± 3.0 (11.4)	130.9 ± 4.1 (15.8)	125.7 ± 2.7 (10.6)	129.3 ± 3.0 (11.8)	.82	**Chocolate**	125.4 ± 2.9 (10.9)	126.8 ± 2.8 (10.3)	124.2 ± 2.2 (8.1)	.79

**Control**	10	135.7 ± 3.9 (12.4)	134.3 ± 4.8 (15.2)	130.8 ± 5. 8 (18.3)	134.0 ± 3.9 (12.3)	.98					

**Median DBP ± SE (SD)**											

**Chocolate**	11	83.6 ± 3.2 (10.6)	83.9 ± 4. 0 (13.4)	84.5 ± 3. 5 (11.6)	80.8 ± 3. 8 (12.5)	.49	**Tomato**	82.8 ± 2.8 (9.3)	82.6 ± 3. 4 (11.3)	79.7 ± 3.7 (12.3)	.91

**Tomato**	15	79.1 ± 1. 9 (7.5)	79.0 ± 2.7 (10.4)	77.5 ± 1.9 (7.4)	79.2 ± 2.2 (8.6)	.98	**Chocolate**	76.4 ± 2.1 (7.7)	77.9 ± 2.2 (8.2)	77.8 ± 1.7 (6.2)	1.0

**Control**	10	77.8 ± 2.7 (8.6)	76.6 ± 3.0 (9.4)	77.3 ± 3.2 (10.0)	77.3 ± 3.0 (9.4)	.94					

P-values were calculated using linear mixed model analysis. Between group differences over time (phases 1 and 2) were not significant (P-values not shown).

### Other characteristics

Weight, body mass index (BMI), abdominal circumference, physical activity, general health, energy and mood levels were assessed at three time points at 0, 8 and 20 weeks (baseline, end of intervention period in phase 1, and end of intervention period in phase 2). No significant changes over time within the three groups were observed (Table 3). Differences in characteristics between groups remained insignificant (data not shown). Not all participants agreed to have their abdominal circumference measured at all time points, therefore mean values are not representative of all participants and changes over time need to be interpreted cautiously.

**Table 3:** Changes over time in selected characteristics of participants

**Allocated group at baseline**	**Dark chocolate**, n = 11				**Tomato extract**, n = 15				**Control**, n = 10		
	**Mean (SD)**				**Mean (SD)**				**Mean (SD)**		

**Time point, wks**	**0**	**8**	**20**	**P-value^a^**	**0**	**8**	**20**	**P-value^a^**	**0**	**8**	**P-value^a^**

**Characteristics**											

Weight, kg	80.3 (17.7)	80.7 (17.9)	80.5 (18.5)	1.0	74.0 (10.3)	73.8 (10.3)	75.3 (9.0)	.96	75.9 (13.7)	75.6 (13.6)	.80

Body mass index	26.9 (6.2)	27.2 (6.9)	27.1 (7.2)	.99	26.2 (3.1)	26.2 (2.7)	26.6 (2.7)	.96	26.2 (5.2)	26.13 (5.1)	.88

Abdominal circumference, cm	85.7 (9.5)	81.9 (10.0)	90.5 (13.0)	(.43)^b^	87.5 (11.5)	87.0 (10.2)	89.8 (12.2)	(.84)^b^	82.2 (9.3)	90.5 (13.0)	(.22)^b^
	
	n = 6	n = 7	n = 4		n = 13	n = 12	n = 8		n = 7	n = 6	

Physical activity ≥ 30 min, d/wk	2.9 (2.0)	3.0 (1.8)	2.3 (0.9)	.99	3.8 (2.5)	3.5 (2.2)	3.0 (2.0)	.61	3.3 (2.5)	3.7 (2.6)	.30

5-point Likert scales											

General health^c^	2.3 (0.9)	2.6 (1.1)	2.4 (0.7)	.78	2.0 (0.8)	1.9 (1.1)	2.3 (1.0)	.92	2.2 (0.8)	2.2 (0.8)	.82

Energy levels^d^	2.5 (0.8)	2.4 (1.2)	2.4 (1.3)	.89	2.3 (1.0)	2.1 (0.9)	2.5 (1.2)	.68	2.3 (0.8)	2.6 (1.2)	.22

Mood^e^	2.1 (0.5)	2.2 (0.8)	2.2 (0.6)	.93	2.1 (0.7)	2. 0 (0.7)	2.1 (0.7)	.86	2.0 (0.7)	2.3 (0.5)	.20

Time points: Week 0 (baseline) and week 8 (end of intervention period of phase 1, parallel RCT); week 20 (end of intervention period of phase 2, crossover)

^a ^P-values of differences over time within a group by linear mixed model analysis

^b ^P-values not based on equal numbers at different time points

^c ^5-point scale with 1 = excellent, 2 = very good, 3 = good, 4 = fair, 5 = poor

^d ^5-point scale with 1 = very energetic, 2 = moderately energetic, 3 = somewhat energetic, 4 = fatigued, 5 = very fatigued

^e ^5-point scale with 1 = very happy, 2 = happy, 3 = up and down, 4 = unhappy, 5 = very unhappy

### Acceptability

At the end of the intervention periods of phases 1 and 2 we assessed acceptability and willingness to continue with treatment long-term with daily consumption of chocolate or a capsule. The two chocolate groups of phase 1 and crossover phase 2 were analysed together, as well as all capsule groups (tomato extract and placebo groups of phase 1 and tomato extract group after crossover in phase 2). Differences in acceptability between the two treatment forms were highly significant (p < 0.0001, effect size 0.9): half of the participants allocated to the chocolate treatment found it hard to eat 50 g of dark chocolate every day and 20% considered it an unacceptable long-term treatment option, whereas all participants found it easy and acceptable to take a capsule each day for blood pressure (Table 4). Unacceptable taste, concerns about fat content, and side effects such as constipation and headache were reported reasons for unacceptability of chocolate as a regular treatment option. The majority of participants stated that they would be willing to continue treatment if it was effective (Table 4). Follow-up of participants three months after the trial revealed that tomato product consumption did not change significantly, although 80% had intended to add more tomatoes to their diet. However, dark chocolate consumption increased after the trial in all groups (Table 4).

**Table 4:** Acceptability of treatment (chocolate or capsules)

	**Dark chocolate groups:**	**Capsule groups:**	**Mean (SD)**	**P-value^a^**
	**70% cocoa, 50 g daily**	**tomato extract (15 mg lycopene) or placebo, 1 capsule daily**		

**Ease of taking supplements^b^**	50% found it hard to eat 50 g dark chocolate daily for 8 weeks	100% found it easy to take one capsule daily for 8 weeks	Chocolate groups: 2.4 (1.3)	
			
			Capsule groups: 1.3 (0.5)	p < 0.0001

**Acceptability^c^**	20% found it unacceptable	100% acceptable	Chocolate groups: 1.9 (1.0)	
			
			Capsule groups: 1.3 (0.5)	p < 0.0001

**Reason for unacceptability**	Unpalatable, too rich, encountered side-effects, such as constipation and headache	N/A		

**Withdrawals**	unpalatable (n = 2)	gastrointestinal upset (n = 1); illness unrelated to study (n = 1)		

**Willingness to continue with treatment after trial^d^**	73% would continue with daily dark chocolate;	100% would continue with capsules;		

	Average dark chocolate consumption increased after the trial compared to baseline: mean increase = 44 ± 194 g/week	80% intended adding more tomatoes to diet;		
		
		35% would rather eat tomatoes than take a capsule		

**Willingness to pay for supplements^d^**	73% would be willing to spend ~A$1.25 for 50 g dark chocolate per day	66% would be willing to spend ~A$1 per capsule per day		

^a^P-values are calculated by student t-test,

^b ^Ease was assessed using a 5-point Likert scale with 1 = very easy, 2 = easy, 3 = sometimes easy, sometimes hard, 4 = hard, 5 = very hard;

^c ^Acceptability was assessed using a 4 = point Likert scale with 1 = very acceptable, 2 = acceptable, 3 = unacceptable, 4 = very unacceptable

^d^Willingness for long-term treatment was estimated by bivariate questions (yes/no)

## Discussion

Our trial did not demonstrate an effect of either dark chocolate or tomato extract on blood pressure in prehypertensive individuals over 8 week intervention periods. However, our trial provided clear insights into acceptability and practicability regarding long-term treatment modalities for blood pressure. While half of the participants expressed difficulty in daily consumption of 50 g dark chocolate and 20% did not consider this a long-term treatment option, all trial participants found it acceptable to take a non-prescription capsule a day for their blood pressure.

Dosages of active components in our trial treatment arms were comparable to earlier controlled trials investigating the effect on blood pressure of either dark chocolate (~750 mg polyphenols daily) [15,16,18-23,26,27] or tomato extract (15 mg lycopene daily equivalent to 4–5 medium size tomatoes) [28]. Previous studies of the use of chocolate on blood pressure reported mixed results: a meta-analysis by Taubert et al. [25] including 5 studies found a significant blood pressure reducing effect of chocolate, however more recent publications include non-significant findings in normotensive and prehypertensive populations [15,16,22]. To date, only a few studies have investigated the effect of tomato extract on blood pressure [28,37]. While Engelhard et al. [28] demonstrated a significant effect of tomato extract in untreated hypertensive individuals, this effect was not apparent in our prehypertensive population using an identical dosage.

It is likely that blood pressure level at the start of treatment (hypertension, prehypertension, normotension) is associated with a treatment's effectiveness. Taubert et al. [24] observed a trend towards a greater treatment effect of smaller doses of chocolate in individuals with higher starting blood pressures, and our recent subgroup meta-analysis of the effect of garlic supplements on blood pressure demonstrated that reductions in blood pressure were clearly correlated with starting blood pressures [36].

Other factors, such as duration of intervention, frequency of blood pressure assessments, and inclusion of washout periods may also influence findings. While most trials investigating the effect of chocolate on blood pressure were of short duration (2 weeks, [15,16,18-23,26,27], a few used longer intervention periods of 6 weeks [16], 8 weeks [15], or 18 weeks [24], and monitored blood pressure at multiple time points during the intervention periods [15,24]. Non-linear fluctuations in blood pressure similar to those observed in our trial were seen over an 8-week period by Allen RR et al. [15]. Furthermore, inclusion of washout periods in our trial revealed a change of blood pressure over time independent of treatment, suggesting that blood pressure median values regressed towards the mean over the 24 week (6 month) study period. The majority of previous trials have not assessed blood pressure changes during washout periods [15,16,18,19,22,24,27].

Our trial had some limitations. First, the relatively small sample size provided inadequate power to detect any differences in blood pressure smaller than 5 mm Hg in SBP or 3 mm Hg in DBP between the groups. These BP levels are similar to estimated minimum BP reductions achievable with lifestyle modifications such as weight reduction (to maintain normal BMI 18.5–24.5), consumption of a diet rich in fruits and vegetables and low in saturated fat, and engagement in daily moderate physical activity (≥30 min, e.g. brisk walking) [1]. However, it is possible that smaller reductions in BP may have long-term cardiovascular health benefits. Second, inclusion of a control group after crossover and follow-up over 24 weeks might have assisted in interpretation of findings. Third, blinding of participants to the chocolate treatment was impractical. Lastly, although we assessed overall polyphenol content of the trial chocolate, we were not in a position to assess specific polyphenol components.

It is debateable whether an initial run-in period should have been included. While the majority of similar trials to date on the effect of chocolate on blood pressure chose a one-week cocoa/flavanol-free run-in period [16,19-24,26], we instead assessed any habitual cocoa or tomato product intake at baseline and throughout the study with the aim to incorporate any individual changes in consumption in the analysis. This study design provided greater generalisability of findings to the whole prehypertensive population seen in routine clinical practice.

Despite some limitations, our trial, for the first time, compared the practicability of using two different treatment modalities (chocolate or capsule) for people with blood pressures in the prehypertensive range. Acceptability and projected long-term compliance of chocolate as a treatment option for blood pressure might be limited, suggesting that chocolate may be a less practical treatment option than capsules or pills. This finding was somewhat unexpected, as recruitment was mainly driven by the interest of the public in a study using chocolate.

While our trial was not designed to assess the effect of chocolate or tomato extract on other cardiovascular factors such as lipid levels or endothelial function, other studies in prehypertensive and normotensive populations have associated chocolate consumption with improved coronary circulation [22], improved endothelial function [10,27], induced nitric oxide dependent vasodilation [11], or a reduction in serum total and LDL cholesterol (in a population with elevated serum cholesterol) [15]. In light of the potential cardiovascular benefits of chocolate or tomato products on factors other than blood pressure, and the potential to forestall progression to hypertension by ongoing treatment (e.g. 2 years) of prehypertension [4], future long-term studies assessing cardiovascular outcomes, tolerance and compliance of a dietary treatment would be of interest.

## Conclusion

Our study did not find a blood pressure reducing effect of 50 g dark chocolate daily or 15 mg lycopene containing tomato extract daily in a small prehypertensive population over a period of 8 weeks. While we cannot rule out cardiovascular health benefits of these dietary treatment options, our study suggests that chocolate may not be an acceptable long-term treatment option for some individuals.

## List of abbreviations

Abdom. circ.: abdominal circumference; BP: blood pressure; BMI: body mass index; CI: confidence interval; d: day; DBP: diastolic blood pressure; g: gram; mg: milligram; min: minutes; mm Hg: millimetre mercury; n: number; RCT: randomised controlled trial; RR: relative risk; SBP: systolic blood pressure; SD: standard deviation; SE: standard error; LDL: low density lipoprotein that transports cholesterol and triglycerides; Wk: week.

## Competing interests

The study was conceived and initiated by the authors. Haigh's Chocolates, Adelaide, LycoRed Ltd, Beer Sheva, Israel, and JA Davey Pty Ltd, Melbourne donated their products at the authors' request. They did not provide funding and were not involved in study design, data collection, analysis or preparation of the manuscript. The authors declare that they have no competing interests.

## Authors' contributions

All authors conceptualised the study and obtained funding. KR managed data acquisition, analysis and interpretation in discussion with the statistician NB and authors ORF and NPS. KR prepared the manuscript with contributions from co:authors. All authors approved the final version.

## Pre-publication history

The pre-publication history for this paper can be accessed here:


